# In Experts, underlying processes that drive visuomotor adaptation are different than in Novices

**DOI:** 10.3389/fnhum.2015.00050

**Published:** 2015-02-10

**Authors:** Christian Leukel, Albert Gollhofer, Wolfgang Taube

**Affiliations:** ^1^Department of Sport Science, University of FreiburgFreiburg, Germany; ^2^Department of Medicine, Movement and Sport Science, University of FreibourgFreibourg, Switzerland; ^3^Bernstein Center Freiburg, University of FreiburgFreiburg, Germany

**Keywords:** expertise, implicit learning, motor learning, prismatic adaptation, skill level

## Abstract

Processes responsible for improvements in motor performance are often contrasted in an explicit and an implicit part. Explicit learning enables task success by using strategic (declarative) knowledge. Implicit learning refers to a change in motor performance without conscious effort. In this study, we tested the contribution of explicit and implicit processes in a visuomotor adaptation task in subjects with different expertise in the task they were asked to adapt. Thirty handball players (Experts) and 30 subjects without handball experience (Novices) participated. Three experiments tested visuomotor adaptation of a free throw in team handball using prismatic glasses. The difference between experiments was that in Experiment 2 and 3, contribution of explicit processes was prevented, whereas Experiment 1 allowed contribution of explicit and implicit processes. Retention was assessed in Experiment 3. There were three main findings: (i) contribution of explicit processes to adaptation was stronger in Experts than Novices (Experiment 1); (ii) adaptation took longer in Experts when preventing contribution of explicit processes (Experiment 2); and (iii) retention was stronger in Experts (Experiment 3). This study shows that learning processes involved in visuomotor adaptation change by expertise, with more involvement of explicit processes and most likely other implicit processes to adaptation in Experts.

## Introduction

The apparent ease with which humans learn new movements or adapt movements they once acquired may suggest that the mechanisms driving changes in behavior are trivial. This is certainly not the case. In fact, the highly complex mechanisms that underlie the various forms of motor learning are still poorly understood. A very popular approach to study motor learning is to induce sensorimotor perturbations (Krakauer, 2009). In visuomotor adaptations, visual sensation is distorted and subjects are required to adapt motor output to reach a predefined goal. This approach allows assessing the contribution of different learning processes based on the subjects’ behavior before, during, and after adaptation.

However, a significant limitation of the experiments performed so far is that the movement experience of the subjects that perform the visuomotor adaptation has not been considered. This refers to the postulation that the strength of contribution of learning processes change with the level of motor expertise. These fundamental changes may influence the way subjects adapt. Fitts and Posner (1967) argued that an inexperienced person performing a motor task (termed Novice in the following) extensively relies on explicit (strategic, i.e., declarative) knowledge. In contrast, in a skilled person (termed Expert in the following), behavior is dominantly controlled by implicit and, therefore, subconscious processes with little contribution of explicit processes. This means, in the model proposed by Fitts and Posner (1967), that the contribution of explicit and implicit processes shifts when a Novice becomes an Expert in a defined motor task, with explicit processes dominating in Novices and implicit processes dominating in Experts.

Consequently, in the present study we asked whether learning processes in Experts are different in contrast to Novices when asked to modify the task they are experienced in. The Experts in our study consisted of experienced handball players and the Novices were age and gender matched students with no experience in handball. The experimental design was a visuomotor adaptation to prism glasses (Helmholtz, 1867) in a standardized free throw in team handball. The contribution of explicit processes to adaptation was assessed by enquiring about the aiming direction of the subjects prior to each throw. This novel task design, separating explicit from implicit learning processes, was recently introduced by Taylor et al. (2014).

## Methods

We tested a total of 60 subjects (mean age 24 years). Thirty of these subjects played handball in German amateur clubs for many years (at least “Regionalliga”, i.e., third division in Germany, or higher). This group is termed “Experts”. In contrast to these handball players, 30 subjects were Novices who had little or no experience in ballgames. All of these 30 subjects had never performed any handball training. The age, gender, size and weight were matched between the two groups. All of the subjects were right-handed (Oldfield, 1971). The study was performed in accordance with the declaration of Helsinki (latest revision in 2008) and run with general approval of the Ethics committee in Freiburg, Germany.

Three different experiments were conducted. In Experiment 1, we tested adaptation of Experts and Novices to a visual displacement by prism glasses. In Experiment 2, we again tested adaptation but this time aimed to prevent explicit processes to contribute to adaptation by providing additional verbal instruction to the subjects (see Section “Experimental Procedures”). Experiment 3 tested for retention of the adapted movement. Nine Experts and eight Novices participated in Experiment 1, 11 Experts and 12 Novices participated in Experiment 2 and 10 Experts and 10 Novices participated in Experiment 3. Experts were comparable with respect to age, gender, height, weight and handball experience in Experiment 1, 2 and 3. This was also the case for the Novices. Note that the number of subjects participating in each of the experiments is attributed to the availability of participants at the time when the experiments were conducted.

### Basic experimental setup

The subjects were tested in a standardized free throw in team handball. The subjects stood 3.5 m in front of a wall with their left foot behind a blue line marked on the floor and the right foot placed laterally behind the left foot. Subjects had to execute regular free throws. An experienced handball player introduced the procedure of a free throw to the Novices before starting the experiment. We gave all subjects 5 min to practice. When throwing the ball, the right hand was raised above the head and behind the trunk. The throw, therefore, started with a backward countermovement of the right arm. After the countermovement, the hand was pushed forward to finally release the ball. Subjects did not see their arm while throwing. We prevented lateral vision, i.e., visual feedback about the arm position that might have biased the adaptation process with the prism glasses, by mounting a visibility screen to the right side frame of the prism glasses. After the throw was completed, the subjects saw where the ball hit the wall and the experimenter handed the ball back to the subjects. Thereafter, they started a new throw. Importantly, the ball was delivered behind the subjects’ body (i.e., without the subject seeing the hand gripping the ball) with the same intention not to bias the adaptation.

We controlled not only for biasing visual feedback but also auditive feedback. Therefore, a softball with the same size and approximate weight as a handball was used because the softball made no noise when hitting the wall. Thus, the subjects did not receive any auditory cues about the location of the ball when hitting the wall.

We marked the center of the wall with a vertical red line and drew two additional blue lines 15 cm to the left and to the right relative to the center. The area within the two blue lines corresponded to the target area, i.e., hitting this area with the ball meant task success. Furthermore, we drew additional black vertical lines (interspaced by 10 cm) to the left and to the right and marked the distance to the center with numbers (in cm) at a height of 1.8 m. Numbers to the left of the center had negative signs, numbers to the right positive signs. These lines and numbers served to quantify the aiming direction of the subjects (see Section “Experimental Procedures”) and also to measure movement errors. Note that the numbers were only provided in Experiment 1 but not in Experiments 2 and 3. The distance to the center of the target was assessed in steps of 5 cm: 10 cm steps when the ball hit one of the vertical lines and 5 cm when the ball hit the wall between two neighboring vertical lines. In preliminary experiments, we recorded movement errors using a digital camera, analyzed the results offline and compared them with the results of an experimenter (not involved in the former analysis) judging the movement errors online. The results were the same. Consequently, we decided that the online analysis is sufficient to determine movement error. The experimenter judging movement errors kept the same position relative to the wall throughout all experiments.

### Experimental procedures

#### Experiment 1

At the beginning, subjects performed 50 baseline trials. Before the first throw, they were verbally instructed to “aim for the target”. We asked whether they understood the instruction and explained it if required. We repeated the sentence “aim for the target” before every 20^th^ throw. During the baseline trials, subjects wore “sham” prism glasses shifting vision non-significantly leftwards (0.2°). “Sham” prism glasses were used to ensure similar conditions throughout the experiment (that all throws were performed with glasses). After the baseline phase, 300 throws were performed with real prism glasses. Prismatic glasses produced a rightward shift of vision of 16°, corresponding to a displacement of approximately 100 cm on the wall. Prism glasses were put on the subjects (and later changed) while they had their eyes closed. Importantly, subjects were not informed about the characteristics of the prism glasses and no information was given about possible strategies to counteract the rightward visual displacement.

For the last 10 throws of the baseline phase and the 300 trials of the subsequent adaptation phase, subjects had to verbalize their aiming direction immediately prior to each throw. Therefore, the subjects were instructed to state immediately before each throw the number they were aiming for. As mentioned, the numbers corresponded to the different lines marked on the wall.

Finally, after 300 throws, we removed the prism glasses and subjects had to continue for 80 trials in the so-called washout phase with the “sham” prism glasses. The behavior (i.e., throwing performance) in this washout phase, also called “aftereffect”, has been used to indicate the contribution of implicit adaptation (Hinder et al., 2008, 2010; Krakauer, 2009).

#### Experiment 2

Experiment 2 was conducted after finishing Experiment 1 and tested the rate of adaptation without contribution of explicit processes. This experiment was similar to Experiment 1 and also contained a baseline, an adaptation and a washout phase. The duration of the adaptation phase in Experiment 2 was shorter than in Experiment 1 (150 compared with 300 trials) as focus was on the rate of adaptation, which does not require stable adapted behavior. The relevant difference in the protocol of Experiment 2 was that explicit adaptation was prevented by instructing subjects to “aim where you see the target” instead of “aim for the target”. This instruction was provided before the first throw and repeated after every 20 trials during baseline, adaptation, and washout phase in order to prevent subjects from using strategies for compensation. When providing the instruction the first time, we asked if subjects understood its meaning and explained it if required.

#### Experiment 3

Experiment 3 was again similar to Experiment 1 except that we prevented contribution of explicit processes as in Experiment 2. However, in contrast to Experiments 1 and 2, there was no washout phase after adaptation in Experiment 3. Instead, subjects performed 100 consecutive no-vision trials directly following adaptation in order to assess retention of the adapted movement. Without vision, learning decays towards baseline behavior. In the no-vision phase, subjects did not see the ball after it left the hand and consequently received no feedback about the movement error. This was made possible by using shutter glasses (PLATO visual occlusion spectacles, Translucent Technologies®, Toronto, Canada) in addition to the prism glasses that subjects’ also wore in the no-vision phase. The shutter glasses were triggered (i.e., blinded) when the arm of the subjects during the throw passed the forehead. Triggering was performed manually by one additional experimenter operating a switch. Vision was restored prior to each throw. Subjects were instructed to inform the experimenter when seeing the impact location of the ball. This was however never the case in the present experiment.

### Data analysis and statistics

The movement error of each throw was recorded and the grand mean was calculated for all subjects in each group. An error to the right with respect to the center of the target was indicated as positive value, and, correspondingly, an error to the left as negative value. Accordingly, the aiming direction that was verbally reported by the subjects was recorded analogous to the movement error.

Learning may be quantified by fitting exponential or power functions (*y* = ax^b^) to the data. However, Taylor et al. (2014) pointed out that this requires that learning is monotonic, which may not be the case in adaptation. The present data confirm this objection as the coefficients of determination were between 0.05 and 0.8, with a low average of approximately 0.25 for the exponential fitting and approximately 0.3 for the power function. Therefore, as proposed by Taylor et al. (2014), data were analyzed using parametrical tests, i.e., repeated-measures analysis of variance (ANOVAs) and Student’s *T*-tests. Levene-tests ensured that the assumption of similar variances between groups to perform ANOVAs was not violated. Greenhouse-Geisser corrected values for ANOVAs are reported in case sphericity of the tested samples was violated.

To prepare the data for statistical comparison, the movement error was binned according to the respective phases (baseline, adaptation, washout trials—Experiment 1 and Experiment 2—and no-vision trials in Experiment 3) in bins of 10 in each subject. This was done for all trials and all three experiments, and for the reported aiming direction in the last 10 trials of the baseline and adaptation phase in Experiment 1. Statistics were performed using SPSS 21 (IBM®, Armonk, NY, USA). Mean and standard error of the mean (SEM) are reported.

## Results

### Experiment 1

#### Movement error

**Figure 1:**
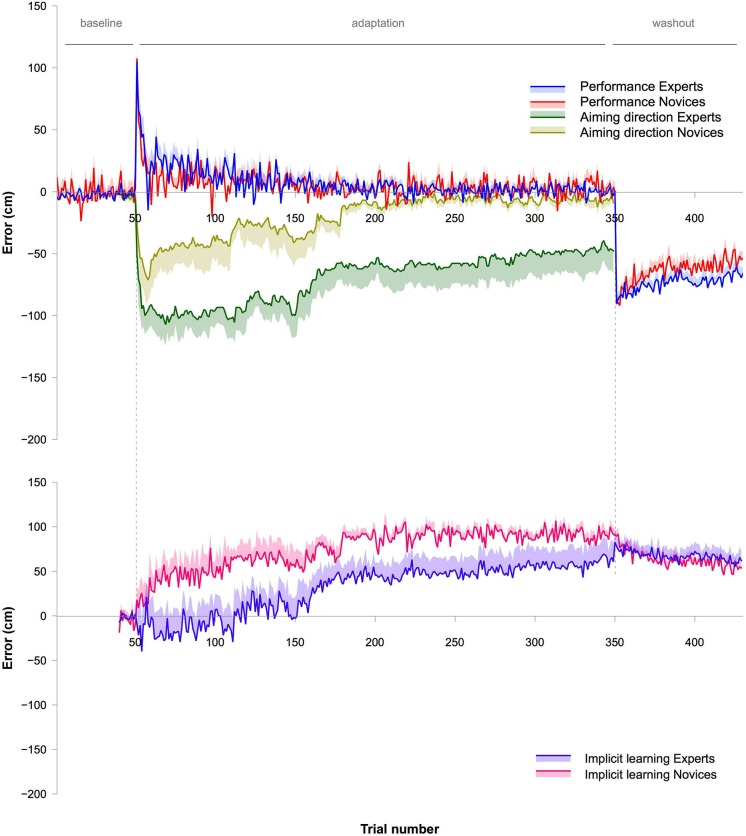
**Upper graph: Depicts the data from Experiment 1**. Blue (Experts) and red lines (Novices) represent the actual movement errors made by the subjects. The shaded areas surrounding the lines represent the standard error of the mean (SEM). Green (Experts) and yellow lines (Novices) represent the aiming direction verbally reported immediately prior to each throw by the subjects. Again, the shaded areas represent SEM. Note that the rate of adaptation was similar between Experts and Novices. However, Experts showed a stronger contribution of explicit processes during adaptation, indicated by the aiming direction. **Lower graph:** Shows the estimated implicit learning (mean and SEM) based on an individual subtraction of the aiming direction from the movement error (see also Taylor et al., 2014). Both, the aiming direction and the movement error, are depicted in the upper part of the graph.

To evaluate whether performance during the adaptation phase differed between Experts and Novices, an ANOVA with the within-subject factor BIN (30 bins, i.e., 300 trials) and the between-subject factor GROUP (Experts vs. Novices) was performed. The ANOVA showed no effect for GROUP (*F*_1,15_ = 0.14, *P* = 0.71, *η*^2^ = 0.01) and no GROUP × BIN interaction (*F*_5.6,84.6_= 1.6, *P* = 0.17, *η*^2^ = 0.09), but a significant effect for TIME (*F*_5.6,84.6_ = 21.1, *P* < 0.001, *η*^2^ = 0.59). These results indicate that subjects successfully adapted with repeated practice, but with no differences in the rate of this adaptation between Experts and Novices (Figures 1, 4, 5).

**Figure 2:**
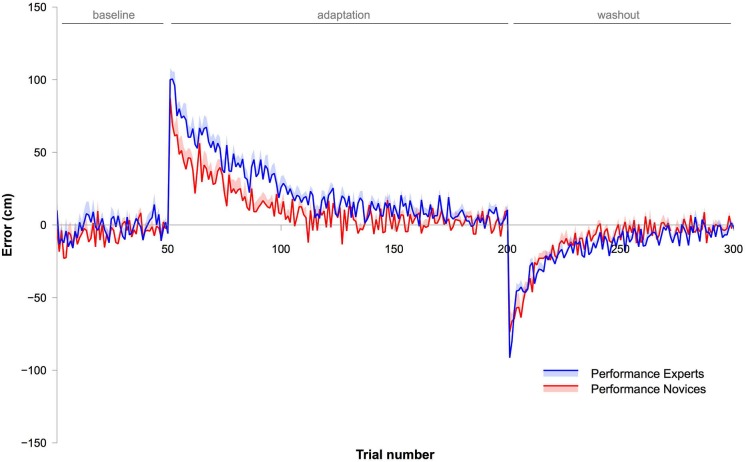
**This graph shows data from Experiment 2**. The blue (Experts) and red lines (Novices) depict movement errors made by the subjects. The shaded areas represent SEM. Note that the rate of adaptation was significantly slower in Experts than in Novices.

**Figure 3:**
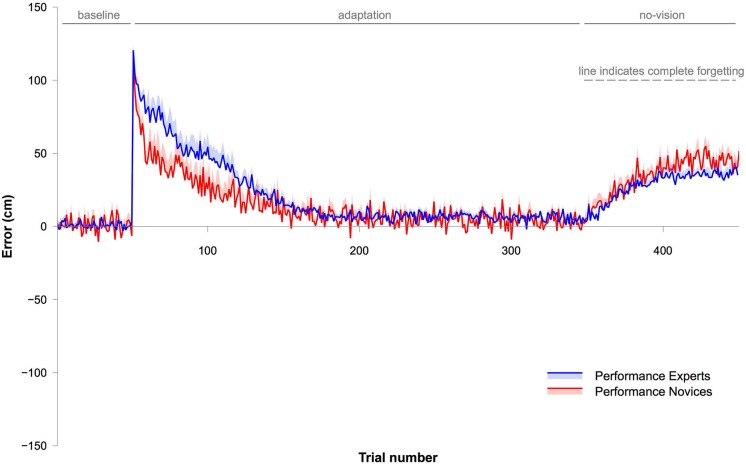
**This graph shows data from Experiment 3 testing retention of the adapted throwing movement**. The blue (Experts) and red lines (Novices) depict movement errors made by the subjects. The shaded areas represent SEM. Note that the rate of adaptation was slower in Experts than in Novices. Most importantly, retention was stronger in Experts, indicated by the no-vision trials. The dashed gray line in the no-vision phase indicates performance if subjects would have completely forgotten the adapted movement, i.e., would have no longer corrected for the displace vision.

**Figure 4:**
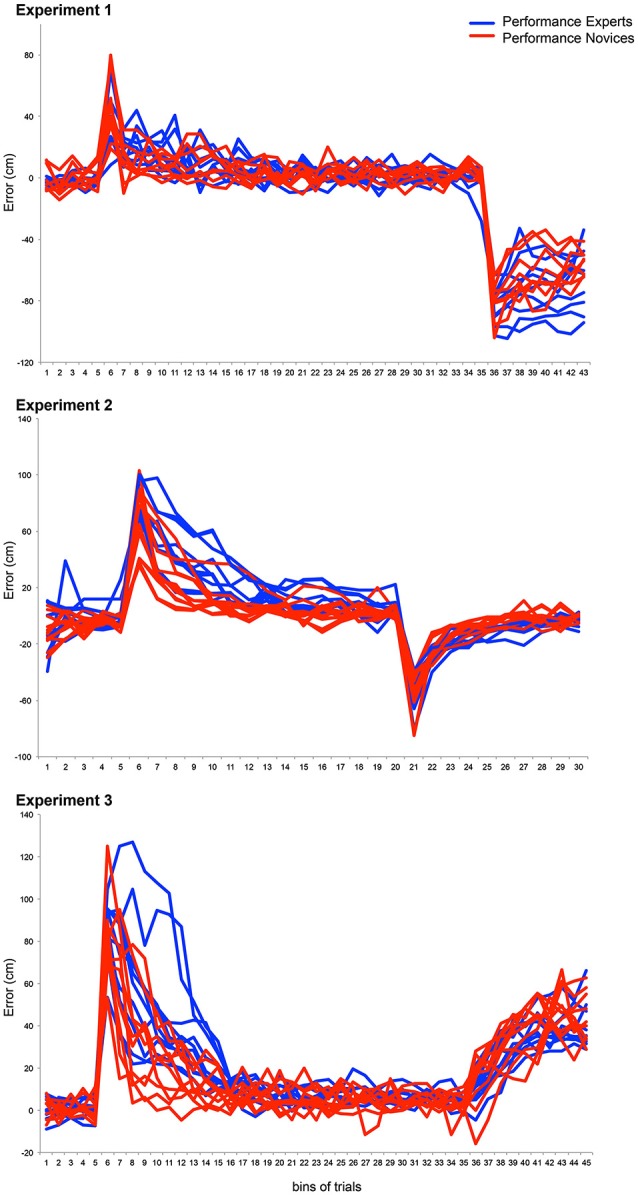
**This graph shows single subject data from Experiments 1 to 3**. The blue (Experts) and red lines (Novices) depict movement errors of bins of trials (i.e., the average of 10 subsequent trials) made by the subjects.

**Figure 5:**
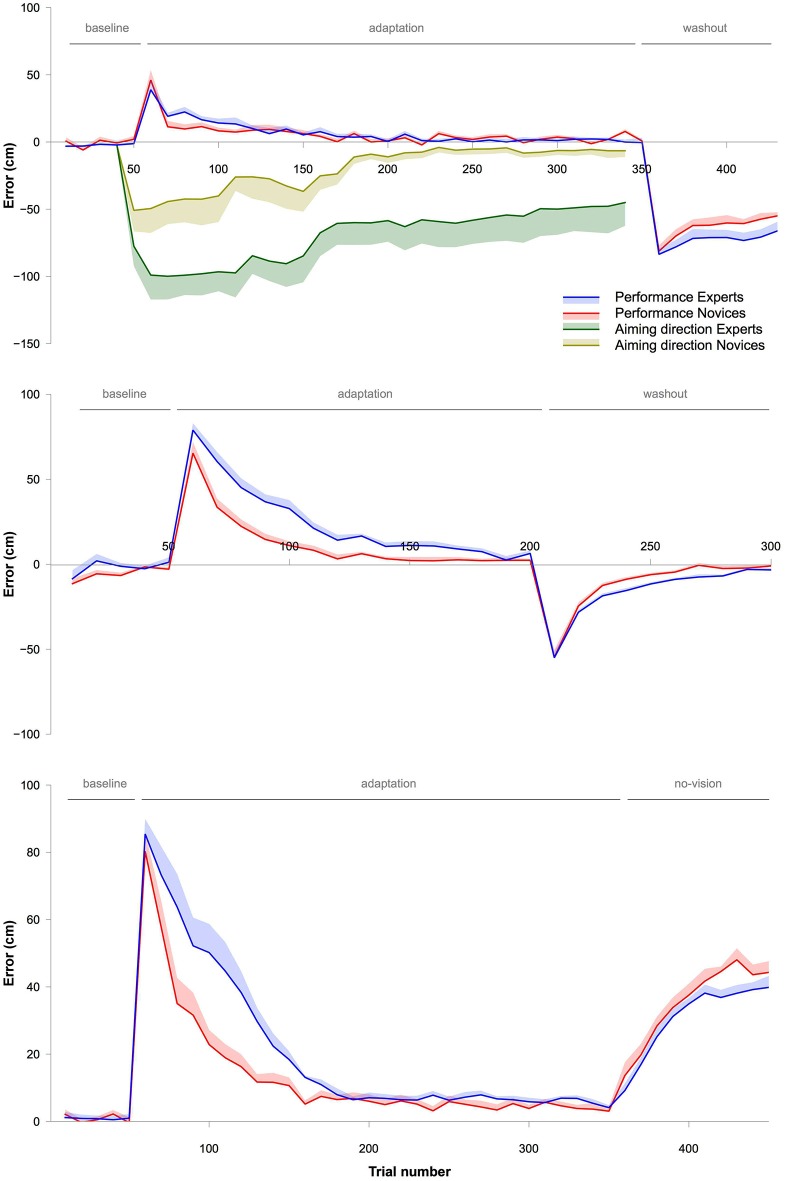
**This graph shows binned movement errors (mean and SEM) that were used for statistical analysis**.

Second, we tested whether groups were different in their baseline behavior just before adaptation and therefore compared the last bin of the baseline phase between groups. An unpaired Student’s *T*-test showed no statistical difference (*P* = 0.24). However, this result does not indicate that performance throughout baseline trials was similar between Experts and Novices. Naturally, Experts should show a much higher consistency of their throws. We tested this consistency by analyzing the individual variation of all baseline throws of Experts and Novices. The variation was significantly smaller in Experts (10.4 ± 0.8 cm) than in Novices (21.5 ± 2 cm) (unpaired Student’s *T*-test: *P* < 0.001), indicating that Experts were indeed more consistent when throwing without prism glasses.

Finally, we tested performance during the washout period between Experts and Novices. Therefore, comparison was made for the first bin of the washout phase between groups. The performance at the beginning of the washout phase has often been used to indicate the strength of implicit processes to adaptation (e.g., Hinder et al., 2008, 2010). There was no significant difference of this initial bin between groups in the present study (unpaired Student’s *T*-test: *P* = 0.71). To analyze whether the rate of de-adaptation was different between Experts and Novices, an ANOVA with the within-subject factor BIN (all 8 bins of the washout phase) and the between-subject factor GROUP (Experts vs. Novices) was performed. The ANOVA revealed no effect for GROUP (*F*_1,15_ = 1.9, *P* = 0.19, *η*^2^ = 0.11), no GROUP × BIN interaction (*F*_7,105_ = 0.98, *P* = 0.45, *η*^2^ = 0.06), but a significant effect for BIN (*F*_7,105_ = 15.5, *P* < 0.001, *η*^2^ = 0.51). This indicates that subjects successfully de-adapted, with no differences between the two groups.

#### Aiming direction

To assess whether the aiming direction differed during adaptation between the two groups, an ANOVA with the within-subject factor BIN (30 bins) and the between-subject factor GROUP (Experts vs. Novices) was conducted. There was a significant effect for GROUP (*F*_1,15_ = 8.4, *P* < 0.05, *η*^2^ = 0.36) and BIN (*F*_2,30.6_ = 6.5, *P* < 0.01, *η*^2^ = 0.3), but no GROUP × BIN interaction (*F*_2,30.6_ = 0.42, *P* = 0.67, *η*^2^ = 0.03). The effect for GROUP indicates that Experts and Novices differed with respect to their aiming direction and hence the contribution of explicit learning processes during adaptation (Figure 1). The effect for BIN further indicates that there were changes in the aiming direction during adaptation. Experts showed a value of around 100 cm at the beginning of the adaptation (note that approximately 100 cm was the actual displacement caused by the prism glasses), Novices showed a value of around 50 cm (Figure 1). Furthermore, Novices turned back to baseline after around 200 trials of the adaptation phase, whereas, at this point in time, Experts still showed values of around 50 cm. Furthermore, in contrast to the Novices, Experts did not return to baseline at all, but remained around 50 cm until the end of the adaptation phase.

The aiming direction was not different between groups just before adaptation in the baseline phase. A Student’s *T*-test showed no significant difference between Experts and Novices (*P* = 0.94).

### Experiment 2

#### Movement error

Differences in adaptation between Experts and Novices were analyzed using an ANOVA with the within-subject factor BIN (15 bins) and the between-subject factor GROUP (Experts vs. Novices). The ANOVA showed a significant effect for BIN (*F*_2.2,46_ = 108.7, *P* < 0.001, *η*^2^ = 0.83), an effect for GROUP (*F*_1,21_ = 10.2, *P* < 0.01, *η*^2^ = 0.33) and a significant GROUP × BIN interaction (*F*_2.2,46_ = 4.1, *P* < 0.05, *η*^2^ = 0.16). These results indicate that all subjects successfully adapted, and that Experts adapted differently than Novices. Figure 2 clearly shows that the rate of adaptation was slower in Experts than in Novices. This is in contrast to the results observed in Experiment 1 where behavior during adaptation was not significantly different between the two groups.

Further, we analyzed whether the movement error of the initial trial of the adaptation phase was different between Experts and Novices, as differences in the initial trials may have influenced the adaptation rate. This was not the case, revealed by an unpaired Student’s *T*-test (*P* = 0.24).

We tested whether group differences in the adaptation phase can be explained by differences in the performance just prior to the adaptation trials. Therefore, the last bin of the baseline phase was compared between groups. Here, an unpaired Student’s *T*-test showed no statistical difference (*P* = 0.20). This result does not indicate that performance throughout all baseline trials was similar between Experts and Novices. Consistency of the throws was tested by analyzing the individual variation of the baseline trials in Experts and Novices. Here, the variation was similar between Experts (17.6 ± 3.1 cm) and Novices (17.8 ± 1.1 cm) (unpaired Student’s *T*-test: *P* = 0.96). This result is in contrast to the result of Experiment 1 where we saw a higher consistency in Experts.

Finally, we compared performance during the washout period between Experts and Novices. Therefore, the first bin of the washout phase was compared between groups. There was no significant difference between groups (unpaired Student’s *T*-test: *P* = 0.56). To indicate whether the rate of de-adaptation was different between Experts and Novices, an ANOVA was performed with the within-subject factor BIN (all 10 bins of the washout phase) and the between-subject factor GROUP (Experts vs. Novices). The ANOVA revealed an effect for GROUP (*F*_1,21_ = 6.2., *P* < 0.05 *η*^2^ = 0.23), an effect for BIN (*F*_2.3,50.2_ = 198.9, *P* < 0.001, *η*^2^ = 0.9) but no GROUP × BIN interaction (*F*_2.3,50.2_ = 1.5, *P* = 0.21). This indicates that subjects successfully de-adapted, and that de-adaptation in Experts was significantly slower than in Novices (see Figures 2, 4, 5).

### Experiment 3

#### Movement error

Differences in adaptation between Experts and Novices were analyzed using an ANOVA with the within-subject factor BIN (30 bins) and the between-subject factor GROUP (Experts vs. Novices). The ANOVA revealed a significant effect for BIN (*F*_2,36.8_ = 69.8, *P* < 0.001, *η*^2^ = 0.8), an effect for GROUP (*F*_1,18_ = 6.6, *P* < 0.05, *η*^2^ = 0.27) and a significant GROUP × BIN interaction (*F*_2,36.8_ = 3.6, *P* < 0.05, *η*^2^ = 0.17). These results indicate that all subjects successfully adapted, but that Experts adapted differently than Novices. Figure 3 clearly shows that the rate of adaptation was slower in Experts than in Novices. A difference in movement error of the first trial of the adaptation phase was not responsible for the altered adaptation rate between the two groups. A Student’s *T*-tests comparing the movement error of the first trial between Experts and Novices revealed no statistical difference (*P* = 0.57).

Consistency of the throws was tested by analyzing the individual variation of the baseline trials in Experts and Novices. As in Experiment 1, the variation was smaller in Experts (7.5 ± 0.6 cm) than in Novices (14.8 ± 2.1 cm) (unpaired Student’s *T*-test: *P* < 0.001). This result, as in Experiment 1, indicates that consistency of throws in Experts was higher than in Novices. In summary, two out of three experiments (Experiments 1 and 3, but not Experiment 2) showed this result. We have no explanation why this was not the case in Experiment 2.

Finally, and most importantly, we tested whether forgetting in the no-vision trials (10 bins) was different between Experts and Novices using an ANOVA with the within-subject factor BIN (all 10 bins of the washout phase) and the between-subject factor GROUP (Experts vs. Novices). The ANOVA revealed an effect for GROUP (*F*_1,14_ = 7.7, *P* < 0.05, *η*^2^ = 0.36), an effect for BIN (*F*_3.9,54.5_ = 59.3, *P* < 0.001, *η*^2^ = 0.81), but no GROUP × BIN interaction (*F*_3.9,54.5_ = 2, *P* = 0.11, *η*^2^ = 0.12). This indicates that the adapted movement was unlearnt in the no-vision phase and that Experts retained the adapted movement better than Novices (see Figures 3, 4, 5). Moreover, we analyzed and compared movement variability during the no-vision trials between the two groups. A Student’s *T*-test revealed a significant difference between Experts (12.6 ± 0.9 cm) and Novices (19.2 ± 1.8 cm, *P* < 0.01), indicating that consistency of the adapted throws was higher in Experts.

### Comparison of adaptation rates between experiments

We were interested in whether adaptation rates of the first few throws differed between experiments. The data (Figures 1–5) suggest that adaptation with explicit and implicit processes in Experiment 1 was faster than in Experiments 2 and 3. To test this, we first calculated differences between movement errors of successive trials for the first 16 trials for each subject, starting with the first trial of the adaptation. This aimed to equalize the initial movement error in the adaptation phase across subjects. These values were analyzed with an ANOVA with the within-subject factor BIN (15 values) and the between-subject factor EXPERIMENT (Experiments 1, 2, and 3). Note that all subjects were included into the analyses without separating them into two groups (Experts and Novices). The ANOVA revealed a significant effect for EXPERIMENT (*F*_2,53_ = 26.2, *P* < 0.001, *η*^2^ = 0.5). To specifically test which of the experiments were different from each other we contrasted them separately using ANOVAs with the with-subject factor BIN (15 values) and the between subject factor EXPERIMENT (either Experiment 1 vs. 2, or Experiment 1 vs. 3, or Experiment 2 vs. 3). Comparing Experiment 1 with Experiment 2 revealed a significant effect for EXPERIMENT (*F*_1,34_ = 28.9, *P* < 0.001, *η*^2^ = 0.46), and the comparison between Experiment 1 and Experiment 3 revealed also a significant effect for the factor EXPERIMENT (*F*_1,35_ = 39.3, *P* < 0.001, *η*^2^ = 0.53). In contrast, the comparison of Experiment 2 and Experiment 3 revealed no significant effect for the factor EXPERIMENT (*F*_1,37_ = 1.88, *P* = 0.18, *η*^2^ = 0.05 ). These results indicate that the adaptation rate of the initial throws was the same in Experiment 2 and 3 but significantly faster in Experiment 1 (Figures 4, 5).

## Discussion

To our knowledge, this is the first study comparing visuomotor adaptation in subjects with different expertise levels of the motor task that had to be adapted. There were two relevant findings. First, contribution of explicit processes in Experts was much stronger than in Novices and, second, adaptation with implicit processes took longer and retention was better in Experts than in Novices.

Explicit processes were assessed via the aiming direction introduced by Taylor et al. (2014). In the present study, the aiming direction of Experts at the beginning of the adaptation phase showed a mean of around 100 cm, and this judgement reflected the actual displacement of the prism glasses of 100 cm. Importantly, subjects were not informed about the characteristics of the visual displacement. In contrast, Novices showed values of around 50 cm. Furthermore, the aiming direction in Novices turned to 0 cm after around 200 trials, whereas Experts still showed values of around 70 cm and this value slightly decreased to around 50 cm but continued until the end of the adaptation phase.

Interestingly, the rate of adaptation and de-adaptation was slower in Experts as soon as contribution of explicit processes was prevented (Experiment 2 and Experiment 3). One may be surprised that it took Experts longer (Experiment 2 and Experiment 3) or equally long (Experiment 1) to adapt than Novices. Intuitively, Experts should be “superior” to Novices and this should be reflected in their behavior, i.e., in our experiment a faster adaptation. Furthermore, as explicit knowledge can be more flexibly applied and therefore may facilitate adaptation (Mazzoni and Krakauer, 2006), our finding of a higher contribution of explicit processes in Experts should speed up adaptation in Experiment 1.

Indeed, Experts in the present study were “superior” to Novices, referring to the higher consistency of their baseline throws, and this consistency is a suitable marker of expertise (Wagner et al., 2012). How can the different behavior between Experts and Novices during adaptation then be explained?

A possibility that would explain our results is that Experts and Novices use different implicit processes during adaptation that are characterized by differences in the learning rate. Previous studies argued that there might exist more than one form of implicit processes that drive visuomotor adaptation (Huang et al., 2011; Shmuelof et al., 2012). These studies discussed a so-called “model-free” learning mechanism that retains learned movements better than a model-based learning mechanisms depending on sensory prediction errors (Miall et al., 1993). The model-free mechanisms implies that this process does not generalize based on learning algorithms but simply reinforces behavior that was previously shown to be successful (Haith and Krakauer, 2013). In force-field adaptation, Smith et al. (2006) provided evidence that at least two processes, both of them model-based, interact: a process that produces rapid changes in motor performance but has poor retention of the learned behavior, and a process that is slower but retains information well. In this sense, it may be that Experts use processes with a slower learning rate but higher retention.

In fact, our results support the view that different implicit learning processes were involved; retention was significantly better in Experts than in Novices in Experiment 3. The rate of adaptation in Experts, on the other hand, was slower when we prevented contribution of explicit processes to adaptation. However, when explicit processes were allowed in Experiment 1, Experts showed similar rates of adaptation and de-adaptation than Novices. The similar rates in Experiment 1 can be explained by the combination of more explicit and slower implicit processes in Experts vs. less explicit and faster implicit processes in Novices. This specific combination of explicit and implicit processes in Experts would therefore not be advantageous over Novices in the sense of an increased speed in adaptation but rather in terms of a better retrieval of the learned movement(s). Furthermore, movement variability in the no-vision (retrieval) phase in Experiment 3 of our study was smaller in Experts than in Novices. This means that a second advantage over better retrieval would be a higher consistency of the adapted (i.e., to the visual displacement) movement. This finding of a higher consistency is similar to results of a previous study, which also investigated behavioral changes based on different forms of implicit learning processes (Shmuelof et al., 2012). Shmuelof et al. argued that a model-free learning process was responsible for the higher consistency.

Although the present findings are restricted to adaptation, one may speculate about fundamental differences of learning processes in relation to motor expertise. We already mentioned in the introduction the “three stages model” by Fitts and Posner (1967), claiming that there is a transition from explicit to implicit processes with increased levels of expertise. Recently, this view was questioned by Stanley and Krakauer (2013). They theoretically argued against the idea that Experts do not use explicit processes: “While it is indeed true that certain motor activities can become habitual or automatic over time, we would again argue that is exactly what does not happen when a motor skill such as tennis or piano is being enacted. In fact the opposite is the case: the (…) athlete is using knowledge of (…) the game to dictate those automatic non-knowledge based components: it is the combination that leads to skilled performance” (Stanley and Krakauer, 2013). The stronger contribution of explicit processes during adaptions in Experts in our study (i.e., the precision in assessing the actual visual displacement and the prolonged utilization in contrast to Novices) argue against Fitts and Posner (1967) and support the argument made by Stanley and Krakauer (2013), that not only implicit but also explicit processes develop with expertise.

An important issue that needs to be discussed concerns the washout period in Experiment 1. In previous studies, the aftereffect at the beginning of the washout phase was argued to reflect the strength of contribution of implicit processes at the end of the adaptation phase (Hinder et al., 2008, 2010). This interpretation of the aftereffect as a measure of the strength of implicit adaptation may be valid for some studies. However, it depends on the experimental design. In our study, we found no differences in the initial movement error made in the washout phase between Experts and Novices. This would indicate that contribution of implicit processes at the end of the adaptation phase was similar between the two groups. However, we show a larger contribution of explicit processes and hence a lower contribution of implicit processes in Experts than in Novices just prior to the washout phase, derived from higher values of the aiming direction. It is unlikely that the strength of contribution of implicit processes is suddenly increased to match that of Novices when transiting to the washout phase. Thus, it is likely that contribution of implicit processes in the initial trials of the washout phase is lower in Experts than in Novices. We explain this by the specific instruction provided to the subjects in our study and the fact of a very salient cue for the visual displacement affecting cognitive judgement. The latter refers to the perceivable removal of the prism glasses in our study in contrast to the hardly perceivable change in cursor position in previous studies (Hinder et al., 2008, 2010). Regarding the instruction, we told the subjects to “aim for the target”. This instruction implies that subjects could apply explicit as well as implicit processes for adaptation and also de-adaptation. With this instruction, there is no convincing argument why subjects should be prevented from that.

A further issue that requires discussion is our claim that contribution of explicit processes was prevented by simply changing instruction to the subjects from “aim for the target” (Experiment 1) to “aim where you see the target” (Experiments 2 and 3). There is certainly no direct evidence that subjects followed our instruction, meaning that contribution of explicit processes was to a large extent prevented in Experiments 2 and 3. However, there is indirect evidence in terms of the adaptation rates in Experiment 1 vs. Experiments 2 and 3. It is known that implicit adaptation requires sufficient time (Mazzoni and Krakauer, 2006; Krakauer, 2009; Criscimagna-Hemminger et al., 2010) but adaptation with declarative knowledge (explicit) not (Mazzoni and Krakauer, 2006; Schween et al., 2014). In line with these observations, the adaptation rate in Experiment 1 of our study was significantly higher than in Experiments 2 and 3.

In conclusion, we found marked differences between Experts and Novices in the contribution of explicit and implicit processes to adaptation. We argue that, in contrast to Novices, Experts use implicit processes that are characterized by slower learning but better retention. To compensate for the slower rate of implicit adaptation, Experts use more explicit processes that are very flexible to change motor output.

## Conflict of interest statement

The authors declare that the research was conducted in the absence of any commercial or financial relationships that could be construed as a potential conflict of interest.
